# Editorial Comment: The outcomes of mini-laparoscopic pyeloplasty in children - brazilian experience

**DOI:** 10.1590/S1677-5538.IBJU.2019.0381.1

**Published:** 2020-01-10

**Authors:** Lisieux Eyer de Jesus

**Affiliations:** 1 Associação Brasileira de Cirurgia Pediátrica Rio de JaneiroRJ Brasil Associação Brasileira de Cirurgia Pediátrica, Rio de Janeiro, RJ, Brasil;; 2 Universidade Federal Fluminense NiteróiRJ Brasil Universidade Federal Fluminense – UFF, Niterói, RJ, Brasil;; 3 Hospital Federal Servidores do Estado Rio de JaneiroRJ Brasil Hospital Federal Servidores do Estado, Rio de Janeiro, RJ, Brasil

The advantages of laparoscopic pyeloplasty (LP) in the treatment of Pediatric obstructive hydronephrosis (less pain, early dismissal from hospital and less scarring/better cosmesis) are already established for school-aged children and adolescents. A preferential role for LP in small children, however, is not yet recognized, and most comparative trials attest on non-inferiority of laparoscopic techniques, mostly because in this age group lumbar or posterior incisions are usually small and frequently there is no need of muscular incision, even in open techniques. This paper reports the results of LP in severe cases of congenital hydronephrosis (2/3 SFU grade 4 cases), with a 18.4% complication rate, including 2 reoperations for reobstruction, two dislocations of the double J stent (one patient with an intra-ureteral stent) and one omental evisceration in the post-operative period (follow up: minimal 13 months, mean 18 months) (1). All children were ≤ 5 years-old and a third of the cohort were children in their first year of life, including a horse-shoe kidney and two patients previously submitted to a pyelostomy. Surprisingly, considering the ages on the cohort, 29% of the cases showed an obstructive polar vessel.

We congratulate the authors for their efforts to progress to minimally invasive techniques, whose advantages are well pointed in the discussion.

LP in small children is certainly feasible (2, 3). This Brazilian group confirms in this observational study that the results are non-inferior to the traditional open access techniques, but, still, proof for advantages over the open procedures are needed in this age group.

Adding to the authors´ remarks about the specific difficulties to establish minimally invasive surgery to treat babies, I would say that in small children the quality of the equipment is fundamental to get the best results, including acceptable duration of anesthesia: sub-optimal laparoscopic equipment implies prolongation of surgery, as well as increased tiredness, technical difficulties and frustration to the operating surgeon.

Concerning public health services, the managers must be aware of the imperative technical needs and also of the need of institutionally organized and financed training to health professionals, in order to allow progress towards the new minimally invasive techniques that are on the horizon. At the end of the day, however expensive an initial investment on minimally invasive surgery may seem to be, the final results are largely compensatory, including from an economical point of view, appearing as shorter hospital stays and less usage of pain medications, with the same good results exhibited by the open techniques.

There are certainly many ways to skin a cat, but some of them may be quicker and the cat might suffer less.
